# *Clostridium sordellii* genome analysis reveals plasmid localized toxin genes encoded within pathogenicity loci

**DOI:** 10.1186/s12864-015-1613-2

**Published:** 2015-05-16

**Authors:** Edward C. Couchman, Hilary P. Browne, Matt Dunn, Trevor D. Lawley, J. Glenn Songer, Val Hall, Liljana Petrovska, Callum Vidor, Milena Awad, Dena Lyras, Neil F. Fairweather

**Affiliations:** Department of Life Sciences, Centre for Molecular Bacteriology and Infection, Imperial College London, London, SW7 2AZ UK; Wellcome Trust Sanger Institute, Hinxton, UK; Department of Veterinary Science and Microbiology, University of Arizona, Tucson, USA; Anaerobe Reference Laboratory, University Hospital of Wales, Cardiff, UK; Veterinary Laboratories Agency, Addlestone, Surrey, UK; Department of Microbiology, Monash University, Clayton, VIC 3800 Australia

**Keywords:** *Clostridium sordellii*, Large Clostridial Cytotoxin, Plasmid, PaLoc

## Abstract

**Background:**

*Clostridium sordellii* can cause severe infections in animals and humans, the latter associated with trauma, toxic shock and often-fatal gynaecological infections. Strains can produce two large clostridial cytotoxins (LCCs), TcsL and TcsH, related to those produced by *Clostridium difficile, Clostridium novyi* and *Clostridium perfringens*, but the genetic basis of toxin production remains uncharacterised.

**Results:**

Phylogenetic analysis of the genome sequences of 44 strains isolated from human and animal infections in the UK, US and Australia placed the species into four clades. Although all strains originated from animal or clinical disease, only 5 strains contained LCC genes: 4 strains contain *tcsL* alone and one strain contains *tcsL* and *tcsH.* Four toxin-positive strains were found within one clade. Where present, *tcsL* and *tcsH* were localised in a pathogenicity locus, similar to but distinct from that present in *C. difficile*. In contrast to *C. difficile*, where the LCCs are chromosomally localised, the *C. sordellii tcsL* and *tcsH* genes are localised on plasmids. Our data suggest gain and loss of entire toxigenic plasmids in addition to horizontal transfer of the pathogenicity locus. A high quality, annotated sequence of ATCC9714 reveals many putative virulence factors including neuraminidase, phospholipase C and the cholesterol-dependent cytolysin sordellilysin that are highly conserved between all strains studied.

**Conclusions:**

Genome analysis of *C. sordellii* reveals that the LCCs, the major virulence factors, are localised on plasmids. Many strains do not contain the LCC genes; it is probable that in several of these cases the plasmid has been lost upon laboratory subculture. Our data are consistent with LCCs being the primary virulence factors in the majority of infections, but LCC-negative strains may precipitate certain categories of infection. A high quality genome sequence reveals putative virulence factors whose role in virulence can be investigated.

**Electronic supplementary material:**

The online version of this article (doi:10.1186/s12864-015-1613-2) contains supplementary material, which is available to authorized users.

## Background

*Clostridium sordellii* is an anaerobic, Gram-positive, spore-forming species of bacterium [1]. It is commonly found in the soil, and can be found in the gut of animals, including humans [2]; it has also been identified in the vaginal microbiota of a small percentage of women [3]. Human *C. sordellii* infections are rare but can cause a variety of serious diseases, including myonecrosis and toxic shock syndrome [4–6]. Infections are most often associated with traumatic injury, surgery, intravenous drug use and gynaecological events (childbirth, abortion and miscarriage). They generally occur in otherwise healthy individuals but have a high mortality rate; wound infections have a mortality rate of ~50 %, while gynaecological infections are almost universally fatal [3]. An association between *C. sordellii* and the abortion-inducing drug combination mifepristone-misoprostol has been noted; misoprostol may facilitate infection by suppressing the activity of the innate immune system [7]. Early symptoms of infection are non-specific, but within hours of presentation most patients develop hypotension and tachycardia that rapidly progresses to multi-organ failure [3]. *C. sordellii* is also an important animal pathogen, causing disease in farm animals including sheep, horses and cattle [8, 9].

The bacteria can produce several toxins and virulence factors, of which lethal toxin (TcsL) and haemorrhagic toxin (TcsH) are considered the most potent [10]. TcsL and TcsH are members of the Large Clostridial Cytotoxin (LCC) family [11] and are closely related to *C. difficile* toxins TcdB and TcdA respectively [12]. Correlation of TcsL production and disease suggests that TcsL is required for *C. sordellii* to induce toxic shock syndrome [10]; indeed, while the *tcsL*^*+*^_*/*_*tcsH*^−^ strain ATCC9714 is rapidly lethal in a mouse model of infection, mutation of *tcsL* results in a complete loss of virulence [13]. Additionally, certain strains of *C. sordellii* lacking LCCs have been associated with less severe infections in humans [14, 15].

Other putative virulence factors produced by *C. sordellii* include neuraminidase (*nanS*), phospholipase C (PLC, *csp*) and, notably, the cholesterol-dependent cytolysin sordellilysin (*sdl*) [16]. An earlier study of a collection of 14 strains isolated from human cadaver tissues revealed that only one strain encoded and produced TcsL, and while all strains encoded the *sdl* gene, only five produced the protein, suggesting that not all strains are able to express their *sdl* genes, at least under the conditions used [16]. This variation in toxin production between strains may well account for the variation in disease type and severity associated with this species.

We have undertaken a genomic analysis of a collection of *C. sordellii* strains derived mainly from cases of clinical or veterinary disease. The majority of strains do not encode *tcsL* or *tcsH,* but all strains encode the putative virulence factors sordellilysin, phospholipase C and neuraminidase. Where *tcsL* or *tcsH* are present, they are localised on a plasmid. A high quality draft genome sequence of strain ATCC9714 reveals numerous other putative virulence factors and will allow future studies on the pathogenesis of *C. sordellii* infection*.*

## Results

### *C. sordellii* strains divide into four clades

**Fig. 1:**
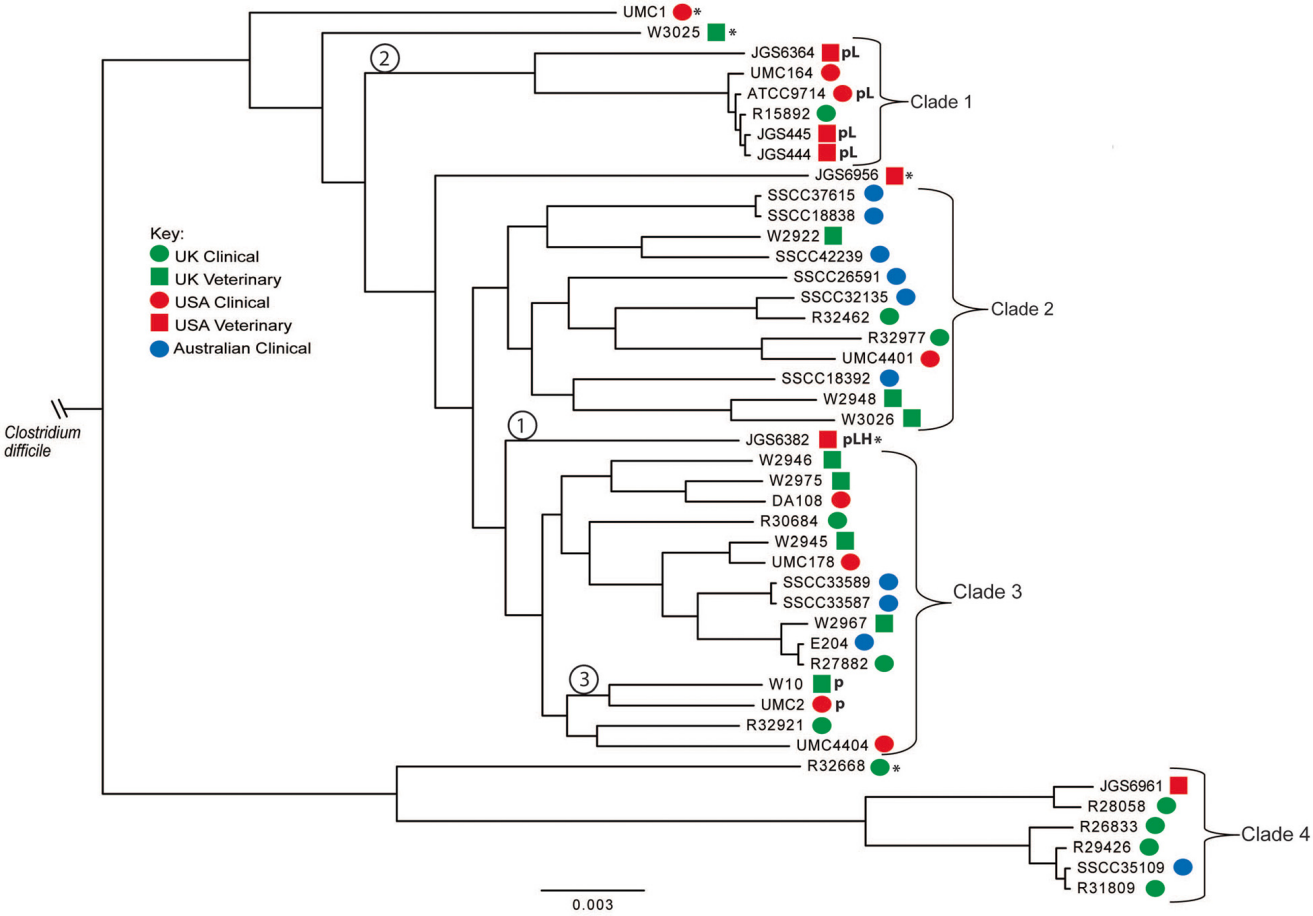
Maximum likelihood phylogeny of 44 strains of *C. sordellii* of clinical and veterinary origin from the UK, USA and Australia. Scale bar denotes nucleotide changes per position. 1000 bootstrap replicates were run resulting in bootstrap support values of greater than 89 % for all nodes. Phylogenetic relationship between *C. difficile* MLST genes and their *C. sordellii* homologues was used to establish the root. *, outlying strains not in a clade; **pL,** strains carrying *tcsL* on pCS1-1 or pCS1-2; **pLH,** strain carrying *tcsL* and *tcsH* on pCS1-3; **p,** strains carrying pCS1-4 or similar plasmid lacking the PaLoc. Encircled numbers indicate hypothesised points of entry of pCS1 type plasmids (see discussion)

A collection of 44 strains of *C. sordellii* was assembled from both clinical and veterinary cases of disease within the UK, USA and Australia (Table 1). Genomic DNA was extracted and sequenced and core gene sequences compared and analysed, generating a phylogenetic tree (Fig. 1). This shows the strains can be divided into 4 primary clades. Five strains appear to be outliers from our selection, not belonging to any clade (indicated with an * in Fig. 1), though it is possible that were more strains to be added these would fall into new, distinct clades. Interestingly, no clade is specific to any country-of-origin, or is specifically associated with either clinical or veterinary disease.

**Table 1:** *C. sordellii* strains used in this study

Strain	Source	Associated infection or pathology	ENA Accession numbers
R32977	ARU (UK)	Knee Replacement	[CELC01000001-CELC01000028[
R32921	ARU (UK)	Death During Pregnancy	[CEKV01000001-CEKV01000024[
R32668	ARU (UK)	Wound Infection	[CEKY01000001-CEKY01000028[
R32462	ARU (UK)	Endo-Cervical Discharge	[CEMX01000001-CEMX01000022[
R31809	ARU (UK)	Traumatic Knee Injury	[CEKW01000001-CEKW01000031[
R30684	ARU (UK)	Calf Abscess	[CELE01000001-CELE01000028]
R29426	ARU (UK)	Sudden Death	[CEMY01000001-CEMY01000040]
R28058	ARU (UK)	Crushed Hand	[CEKZ01000001-CEKZ01000028]
R27882	ARU (UK)	Knee Amputation	[CELB01000001-CELB01000031]
R26833	ARU (UK)	Blood Culture from Diabetic	[CELG01000001-CELG01000030]
W2967	VLA (UK)	Veterinary Isolate	[CELJ01000001-CELJ01000026]
W10	VLA (UK)	Veterinary Isolate	[CELH01000001-CELH01000051]
W2922	VLA (UK)	Veterinary Isolate	[CELK01000001-CELK01000153]
W2945	VLA (UK)	Veterinary Isolate	[CELI01000001-CELI01000027]
W2946	VLA (UK)	Veterinary Isolate	[CENA01000001-CENA01000080]
W2948	VLA (UK)	Veterinary Isolate	[CELF01000001-CELF01000026]
W2975	VLA (UK)	Veterinary Isolate	[CEKX01000001-CEKX01000030]
W3025	VLA (UK)	Veterinary Isolate	[CELA01000001-CELA01000022]
W3026	VLA (UK)	Veterinary Isolate	[CELD01000001-CELD01000043]
JGS444	ISU (USA)	Myonecrosis, Bovine	[CDNJ01000001-CDNJ01000028]
JGS445	ISU (USA)	Myonecrosis, Bovine	[CDNU01000001-CDNU01000031]
JGS6382	ISU (USA) [14]	Myonecrosis, Bovine	[LN681234-LN681235]
JGS6956	ISU (USA)	Veterinary Isolate	[CDNN01000001-CDNN01000034]
JGS6961	ISU (USA)	Veterinary Isolate	[CDNI01000001-CDNI01000027]
ATCC9714	ATCC (USA) [1]	Oedema	[LN679998- LN680000]
DA108	UMich (USA) [15]	Post-Partum Endometritis.	[CDNR01000001-CDNR01000036]
SSCC33589	UWA (AUS)	Blood Isolate	[CDNV01000001-CDNV01000026]
SSCC42239	UWA (AUS)	Blood Isolate	[CDNH01000001-CDNH01000029]
SSCC26591	UWA (AUS)	Blood Isolate	[CDNX01000001-CDNX01000035]
SSCC37615	UWA (AUS)	Blood Isolate	[CDNO01000001-CDNO01000031]
SSCC18838	UWA (AUS)	Blood Isolate	[CDNK01000001-CDNK01000030]
SSCC18392	UWA (AUS)	Blood Isolate	[CDNE01000001-CDNE01000027]
SSCC35109	UWA (AUS)	Blood Isolate	[CDNF01000001-CDNF01000036]
SSCC33587	UWA (AUS)	Blood Isolate	[CDNW01000001-CDNW01000028]
SSCC32135	UWA (AUS)	Blood Isolate	[CDNQ01000001-CDNQ01000031]
UMC1	OU (USA) [16]	Allograft Isolate	[CDNM01000001-CDNM01000035]
UMC2	OU (USA) [16]	Allograft Isolate	[CDLK01000001-CDLK01000054; LN681233]
UMC164	OU (USA) [16]	Allograft Isolate	[CDPO01000001-CDPO01000024]
UMC178	OU (USA) [16]	Allograft Isolate	[CDNP01000001-CDNP01000032]
UMC4401	OU (USA) [16]	Allograft Isolate	[CDNS01000001-CDNS01000027]
UMC4404	OU (USA) [16]	Allograft Isolate	[CDNY01000001-CDNY01000030]
E204	MU (AUS)	Clinical Isolate	[CDNL01000001-CDNL01000025]
R15892	ARU (UK)	Clinical Isolate	[CEKU01000001-CEKU01000032]
JGS6364	ISU (USA)	Myonecrosis, Bovine	[CDLJ01000001-CDLJ01000026; LN681232]

Strains were sourced as indicated: ARU, Anaerobe Reference Unit, Cardiff, UK; VLA, Veterinary Laboratories Agency, Weybridge, UK; ISU, Iowa State University, Ames, IA, USA; ATCC, American Type Culture Collection, Manassas, VA, USA; UMich, University of Michigan, Ann Arbor, MI, USA; UWA, University of Western Australia, Crawley, WA, Australia; OU, University of Oklahoma, Oklahoma City, OK, USA; MU, Monash University, Melbourne, VIC, Australia. The associated pathology or infection is given where known and is human unless otherwise stated.

### The majority of *C. sordellii* strains do not encode the Large Clostridial Cytotoxins TcsL and TcsH

When analysing the initial sequence assemblies we noticed that few strains appeared to contain the genes encoding the LCCs TcsL and TcsH. Five strains contain the *tcsL* gene (ATCC9714, JGS444, JGS445, JGS6382 and JGS6364), and of these only JGS6382 also contains *tcsH*. The four *tcsL*^*+*^*/tcsH*^*−*^ strains are closely related, all found in Clade 1 of our phylogenetic tree, and all contain identical 5’ fragments of the *tcsH* gene, as previously reported for ATCC9714 [17]. No strain contains *tcsH* without *tcsL*. PCR using primers specific for *tcsL* or t*csH* confirmed the presence of the toxin genes only in these genomes (data not shown). Strains encoding *tcsL,* or *tcsL* and *tcsH*, are indicated by **pL** or **pLH**, respectively in Fig. 1 (and see below). Intriguingly, *tcsL* was absent from strain UMC164. UMC164 is part of clade 1 and therefore closely related to all four *tcsL*^*+*^*/tcsH*^*−*^ strains, and a previous analysis in 2006 indicated that it, too, was *tcsL*^*+*^*/tcsH*^*−*^ [16]. UMC164 appears therefore to have lost the *tcsL* gene upon culturing in the laboratory.

### The *C. sordellii* LCC genes are located on one of two plasmids found in ATCC9714

**Fig. 2:**
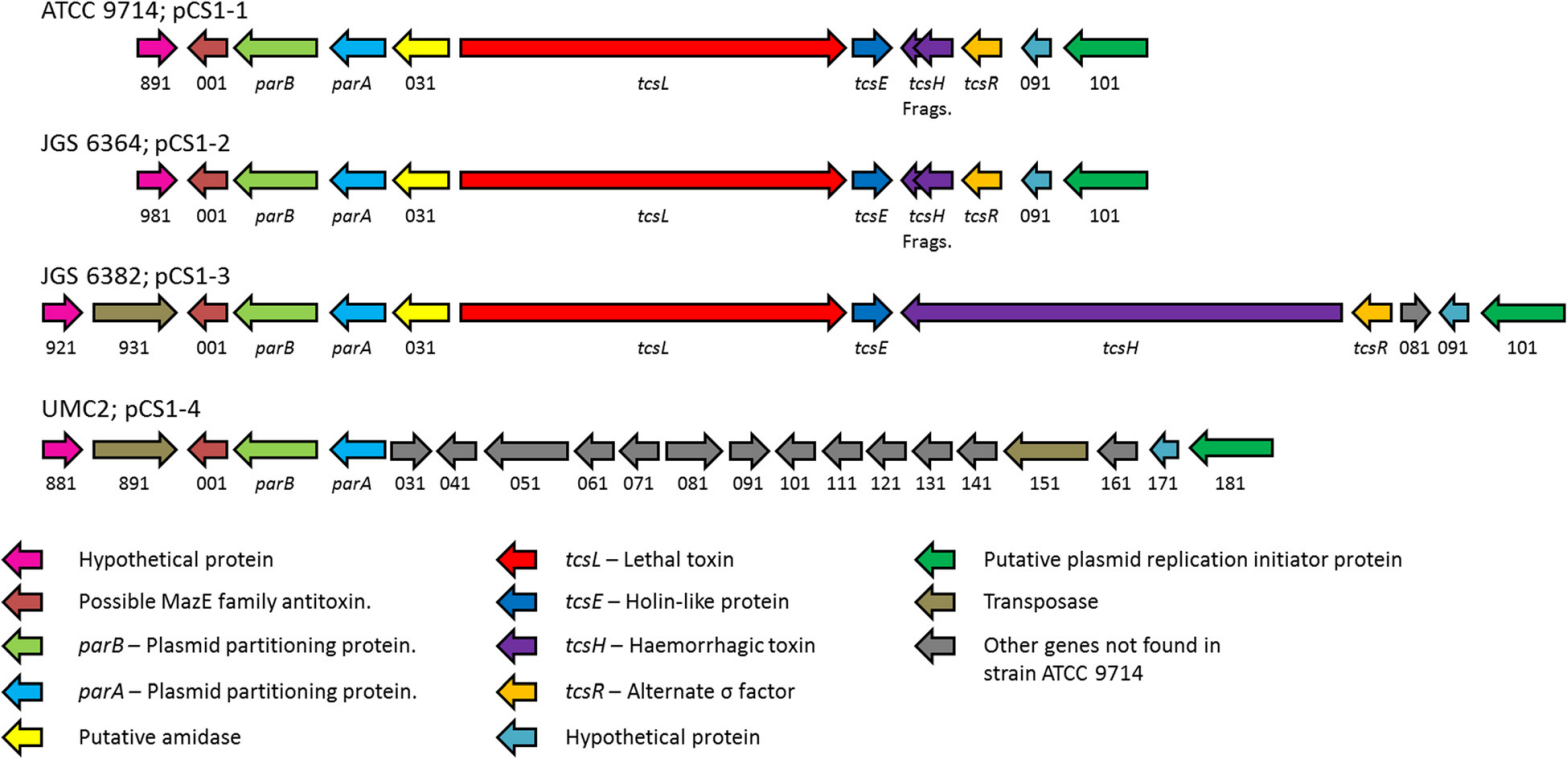
pCS1-type plasmids found in *C. sordellii* strains. PCR was used to confirm the circular nature of these plasmids. Only the regions encompassing the PaLoc and the surrounding region are shown. ORFs are coloured according to function. The fragments of *tcsH* present in pCS1-1/pCS1-2 do not encode any functional toxin component. Plasmids present in strains JGS444 and JGS445 were not assembled but are likely to be highly similar to pCS1-1 (data not shown)

After initial optical mapping of the ATCC9714 genome, it was determined that considerably more sequence remained unmapped than gaps were estimated to remain in the chromosome, suggesting that some of this sequence was localized on extra-chromosomal elements. A large contig of 103.8 kb of unmapped sequence was assembled which could not be optically mapped to the chromosome. PCR demonstrated the circular nature of this element, which we named plasmid pCS1-1. pCS1-1 contains *tcsL*, the alternative sigma factor *tcsR* and the presumed holin protein *tcsE*, present in a pathogenicity locus (PaLoc) in the relative orientations described previously [17]. The PaLoc region of pCS1-1 and the surrounding genes are shown in Fig. 2. pCS1-1 also encodes several proteins likely required for plasmid replication: a replication initiation protein (ATCC9714PCS11_00101), the plasmid-partitioning proteins ParA (ATCC9714PCS11_00021) and ParB (ATCC9714PCS11_00011), a putative topoisomerase (ATCC9714PCS11_00271), a RecA recombinase (ATCC9714PCS11_00141), a resolvase (ATCC9714PCS11_00561) and a helix-destabilising single-stranded DNA-binding protein (ATCC9714PCS11_00391). pCS1-1 also encodes a Type IV Secretion-System DNA conjugation protein (ATCC9714PCS11_00311) and a Type IV Secretion-System coupling DNA-binding domain protein (ATCC9714PCS11_00351), which may aid or enable conjugative transfer of the plasmid. Also found is a secreted collagen-binding protein (ATCC9714PCS11_00241), and a second copy of sortase (one is present on the chromosome) which may anchor certain plasmid-encoded secreted proteins to the cell wall.

An equivalent plasmid termed pCS1-3 was found in JGS6382, the only strain in our collection that carries *tcsH*. After assembly of pCS1-3 by PCR, we found that the genes *tcsL, tcsH* and *tcsE* are arranged identically to those in the *tcsL*^*+*^*/tcsH*^*+*^ strain VPI9048 (Fig. 2) [17]. Thus the *tcsL* and *tcsE* genes are in the same orientation, but *tcsH* and *tcsR* are encoded on the opposite strand. This orientation of the PaLoc genes is different to that found in *C. difficile* where the homologous PaLoc genes *tcdR, tcdB, tcdE* and *tcdA* are in the same orientation. No homologue of *tcdC*, a negative regulator of toxin expression in *C. difficile* [18] was found in any of the *C. sordellii* isolates in our study. Though pCS1-3 is a similar size to pCS1-1 (106 kb) and contains a similar number of ORFs (94 compared to 90 on pCS1-1) it contains several differences in addition to the presence of full-length *tcsH* within the PaLoc. 29 ORFs are absent from pCS1-3 relative to pCS1-1, including 2 universal stress proteins and all genes encoding anaerobic sulphite reductase subunits. However, 32 ORFs are present on pCS1-3 that are not found on pCS1-1, including an additional copy of a resolvase and three transposase genes. Analysis of the sequence of *C. sordellii* VPI9048 [17] reveals that most ORFs from pCS1-3 are also present, suggesting that VPI9048, and potentially other *tcsH*^*+*^ strains, contain a highly related plasmid.

**Fig. 3:**
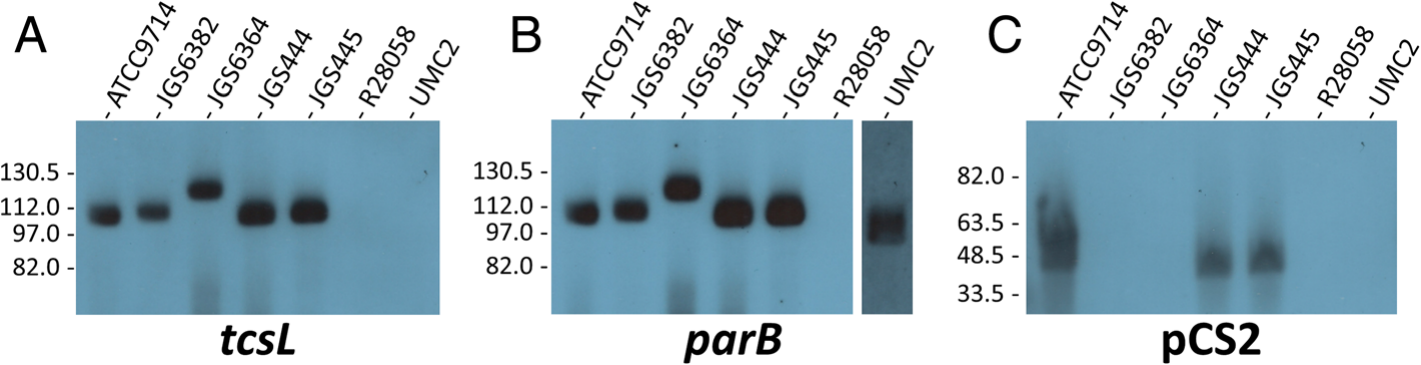
Southern hybridisation analysis of pulsed-field gels to confirm the presence of pCS1- and pCS2-type plasmids in *C. sordellii* strains. **(A)** A blot of a pulsed-field gel using *C. sordellii* genomic DNA-containing agarose plugs digested with *Blp*I (to linearise pCS1-type plasmids), hybridised with a *tcsL* specific probe; **(B)** the blot from panel A was stripped and reprobed with a probe specific for *parB* from pCS1-1; **(C)** a blot of a separate pulsed-field gel using purified uncut *C. sordellii* genomic DNA, hybridised with a probe specific for ATCC9714PCS2_00141 from pCS2. The migration of molecular size markers is indicated on the left, and the *C. sordellii* isolate corresponding to each lane is indicated above

Visual examination of the genomes of the *tcsL*^*+*^/*tcsH*^−^ strains JGS444 and JGS445 suggests that they each carry plasmids highly similar to pCS1-1, though these plasmids were not fully assembled by PCR. Examination of the genome of the *tcsL*^*+*^/*tcsH*^*−*^ strain JGS6364 showed the presence of a plasmid similar to pCS1-1; however, pulsed field gel electrophoresis showed the plasmid in JGS6364 to be noticeably larger than pCS1-1 (Fig. 3), which prompted us to assemble the plasmid by PCR to examine the differences between it and pCS1-1. We designated the plasmid present in JGS6364 as pCS1-2 which, at 117.3 kb, is 13.5 kb larger than pCS1-1. The arrangement of genes within the PaLoc of pCS1-2 was identical to in pCS1-1 (Fig. 2), but several major differences were seen elsewhere on the plasmid. Several genes are lost relative to pCS1-1, including ones encoding a transcriptional regulator and a cold shock protein. Several genes are present which are not found on pCS1-1, including 23 found on a 21 kb insertion. These include various putative lantibiotic biosynthesis genes and a putative lantibiotic-binding component of an ABC transporter. It would be of interest to determine if JGS6364 does indeed produce a lantibiotic, because as far as we are aware lantibiotic synthesis has not previously been identified in the Clostridia.

Two strains in our collection that lack *tcsL* and *tcsH* (UMC2 and W10, indicated with a **p** in Fig. 1) were also found to carry plasmids similar to pCS1-1. The 100 kb plasmid in strain UMC2 was assembled by PCR and designated pCS1-4. pCS1-4 is highly similar to the pCS1 plasmids (Fig. 2), however the PaLoc genes are absent and in their place a series of genes is present including a transposase. Interestingly, in pCS1-3 and pCS1-4, upstream of a conserved putative MazE family antitoxin gene a second transposase gene is found.

During manual assembly of the ATCC9714 genome a second circular element, pCS2, was also identified. pCS2 (37.1 kb) is smaller than pCS1-1 and its relatives, and contains 52 ORFs. pCS2 is clearly phage-derived, with 20 of its 52 ORFs annotated as phage genes, including multiple genes encoding structural phage components. It also contains two genes potentially involved in replication (*soj*/*parA* (ATCC9714PCS2_00031) and a replication initiator protein (ATCC9714PCS2_00011)). Whether this element represents a phage or plasmid is unknown.

### Confirmation of plasmid presence by PFGE and Southern blot analysis

Southern hybridisation analysis of pulsed-field gels was performed to confirm the presence of pCS1-type plasmids among *C. sordellii* isolates (Fig. 3). Examination of the genome sequences of the five toxigenic *C. sordellii* isolates and UMC2 indicated the presence of only a single *Blp*I restriction site on all pCS1-type plasmids. To linearise and therefore better visualise the pCS1-type plasmids, *C. sordellii* genomic DNA-containing plugs were digested with *Blp*I before being subjected to pulsed-field gel analysis. All hybridisation bands observed for *BlpI* digested samples in both the *tcsL* and *parB* blots (Fig. 3) were also seen in the uncut samples (data not shown), confirming that the bands shown in Fig. 3 represent linearised versions of the pCS1-type plasmids. In the *tcsL* blot, a band corresponding to the size of the pCS1-1 plasmid (103 kb) can be seen (Fig. 3A). Bands of equal or higher molecular weight are evident from other toxigenic *C. sordellii* isolates, however, no bands were observed for the non-toxigenic isolates R28058 and UMC2. The size of the pCS1-type plasmids in JGS444 and JGS445 appears to be equal to that of the plasmid in ATCC9714, supporting genome sequence data that indicates these strains carry plasmids highly similar to pCS1-1. The band corresponding to pCS1-3 in JGS6382 appears to be of slightly higher molecular weight than that of pCS1-1, which agrees with the size of pCS1-3 (106 kb) based on genome sequence analysis. The band corresponding to pCS1-2 from JGS6364 is of higher molecular weight than all other pCS1-type plasmids, as discussed earlier. An identical hybridisation pattern can be seen for the *parB* blot in comparison to the *tcsL* blot for the toxigenic *C. sordellii* isolates and the negative control R28058, however, a band can also be seen in the *parB* blot for UMC2 (Fig. 3B), confirming the presence of plasmid pCS1-4 in this strain. A probe for a locus on the pCS2 plasmid (ATCC9714PCS2_00141) was used to confirm the presence of this plasmid in ATCC9714 and to determine if it is present in the other *C. sordellii* isolates tested. A band can be seen in uncut ATCC9714 genomic DNA in the pCS2 blot, indicating that this plasmid is present in this strain (Fig. 3C). Similar bands can be observed from both JGS444 and JGS445, indicating that pCS2 or a related plasmid is also present in these strains, which may be expected as these isolates are phylogenetically related to ATCC9714 (Fig. 1). Indeed, visual analysis of all 44 genome sequences suggests pCS2 is also found in the closely related clade 1 strains UMC164 and R15892. The pCS2 probe did not hybridise to the genomic DNA from any of the remaining *C. sordellii* isolates in the panel of strains tested indicating that this plasmid is not present among these strains.

### The ATCC9714 genome

The sequence of strain ATCC9714 was refined, producing a high quality reference genome. 3282 ORFs are annotated on the chromosome, together with 90 on pCS1-1 and 52 on pCS2 giving a total of 3424. This is in line with other *Clostridial* species – significantly more than *C. perfringens* or *C. tetani* (strains 13 and E88 respectively have approximately 2660 and 2580 chromosomal ORFs) but fewer than *C. difficile* strain 630, which has approximately 3680. ATCC9714 contains the fewest ORFs of the strains in our collection, the most being 3957 ORFs in strain UMC2, and the average being 3459 ORFs. However, these figures are preliminary, as the genomes other than ATCC9714 have undergone little improvement to reduce contig number, which would almost certainly reduce the number of ORFs identified. Further analysis would also allow the identification and assembly of plasmids.

### Putative virulence factors

168 putative secreted proteins were identified in the ATCC9714 genome (Additional file 1), six of which are encoded by pCS1-1 and one by pCS2. Of the 168 ORFs, 66 are predicted to encode lipoproteins, including one encoded on pCS1 and a second on pCS2. There are also nine predicted cell wall proteins, which contain 3 adjacent S-layer homology (SLH) domains, which can non-covalently attach proteins to the cell wall [19]. This attachment requires pyruvylation of the cell wall by the protein CsaB [19], which is encoded on the chromosome (ATCC9714_28031). The *csaB* gene is located just downstream of two SLH domain-containing genes, ATCC9714_28071 (function unknown) and ATCC9714_28081 (a putative amidase). Based on their location adjacent to *csaB*, by analogy to other species, e.g. *Bacillus anthracis* [19], these SLH-domain proteins might be constituents of an S-layer.

Four probable exotoxins were identified: a secreted collagenase *colA* (ATCC9714_10061), the cholesterol-dependent cytolysin (CDC) *sdl* (sordellilysin) (ATCC9714_18311), the neuraminidase *nanS* (ATCC9714_16161) and phospholipase C (*csp*, ATCC9714_31321). The *sdl* gene is highly conserved between all strains sequenced (≥97 % protein sequence identity). The encoded protein shows strong similarity to perfringolysin O from *C. perfringens*, and all residues identified as being of functional or structural importance in perfringolysin O [20, 21] are conserved in sordellilysin, including the undecapeptide. It has previously been shown that sordellilysin functions as a CDC, but that not all strains containing the *sdl* gene produce SDL under standard conditions. Such strains include ATCC9714, UMC164 and UMC178, all part of this collection [16].

NanS is a homologue of the small *C. perfringens* neuraminidase NanH [22]. Multiple catalytically and structurally important residues and motifs have been identified, including four structurally important ‘Asp-Boxes’, all of which are conserved in the ATCC9714 NanS. One important difference between *C. sordellii* NanS and *C. perfringens* NanH is that NanH is located in the cytoplasm, while NanS possesses a signal sequence and has been shown to be secreted, and indeed to cause/contribute to the leukemoid reaction characteristic of severe *C. sordellii* infection [23]. NanS is extremely well conserved between all 44 strains in our collection (≥97 % protein sequence identity); however, two strains, the closely related SSCC37615 and SSCC18838 from clade 2, possess a G= > A point mutation resulting in a Asp162Asn mutation. Asp162 is the equivalent of NanH Asp144, which has been shown to be necessary for correct folding of the protein [22]. NanS produced by these strains may therefore not be fully functional.

Csp is closely related to several Clostridial phospholipases C, including the *Clostridium bifermentans* phospholipase C and *C. perfringens* α-toxin. *C. sordellii* ATCC9714 Csp has been shown to be enzymatically active, but less active than α-toxin, less haemolytic and, alone, to be non-toxic to mice [24]. All known residues essential for enzymatic activity and structural maintenance are conserved within the *C. sordellii* protein [24]. Strains have previously been identified which do not produce Csp, although the majority do [16]. Four closely related strains from clade 4 (R26833, R29426, SSCC35109 and R31809) all possess a T= > A point mutation in codon 50, resulting in the formation of a premature stop codon, which will preventing production of Csp. JGS6382 has an N-terminal truncation resulting in the absence of a signal sequence, meaning it may produce Csp but will not secrete it. All other strains are predicted to produce functional Csp, though JGS6956 Csp has a slight C-terminal truncation. The C-terminus of α-toxin is believed to bind Ca^2+^ ions [25]; the truncation found in JGS6956 Csp would likely render it unable to bind Ca^2+^, which may affect its function.

We are unaware of any prior studies on ColA, the *C. sordellii* collagenase. Comparison with ColG from *C. histolyticum*, the only Clostridial collagenase for which a structure is available, shows some similarity (33 % sequence identity) and the conservation of several important residues, including all those which bind the catalytic Zn^2+^ ion and others important in substrate recognition. However, mature ColA is 79 amino acids shorter than ColG, and the PKD-like domain, important in ColG substrate binding [26], is entirely absent from ColA, raising questions as to how it binds its substrate. However, ColA is well conserved between all strains of *C. sordellii* in our collection (≥95 % sequence identity).

Two putative virulence factors were identified which have predicted functions in immune evasion. ATCC9714_13801 encodes aureolysin, a secreted metalloprotease first identified in *Staphylococcus aureus* which cleaves complement protein C3, preventing complement activation during infection [27], and the anti-microbial peptide LL-37 [28], aiding evasion of the host immune system during infection. ATCC9714_09801 contains a Mac-1 domain, which is found in the *Streptococcus pyogenes* protein IdeS and contains a protease function which specifically cleaves human IgG, aiding immune evasion [29].

Two putative adhesins were also identified on the chromosome. The first is a secreted collagen-binding protein of >140 kDa (ATCC9714_22821/22831), containing at least 7 CnaB repeats. Due to the length and repetitive nature of this gene we were unable to fully sequence it, so it is split across two contigs. The second adhesin is encoded by another large gene (ATCC9714_14191) which contains two discoidin domains; in eukaryotes discoidin domain receptors (DDRs) bind collagen [30]. ATCC9714 also contains two Type IV Pili (T4P) gene clusters (ATCC9714_01091-ATCC9714_01201 and ATCC9714_02121-ATCC9714_02151). T4P are able to promote adhesion both between bacterial cells in biofilms and between bacterial and mammalian cells, and thus commonly act at as virulence factors [31]. These putative adhesins and T4P clusters are conserved in every strain sequenced, as are genes encoding a full flagella apparatus.

It has previously been shown that many, but not all, strains of *C. sordellii* possess urease activity [32, 33]. Urease is a nickel-containing metalloenzyme that hydrolyses urea into ammonia and carbonic acid. It acts as a virulence factor in several bacterial and fungal pathogens, including *Helicobacter pylori* and *Klebsiella* species [34], though there is no evidence that urease acts as a virulence factor in *C. sordellii*. We found complete urease operons in all strains in our collection except SSCC26591. The operon comprises 8 genes, *ureABCIEFGD*, and is located on the chromosome immediately upstream of *nanS* (ATCC9714_16171 – 16241). To our knowledge no other *Clostridia* carry chromosomal urease genes, though a small minority of strains of *C. perfringens* are known to carry urease genes borne on a plasmid [35]. The urease enzyme itself comprises UreABC proteins that initially form an apoenzyme that is activated by the UreDFG complex. UreE is a Ni-binding chaperone and UreI is a urea transporter. All genes are highly conserved between strains (≥87 % protein sequence conservation from every gene). UreC contains the enzyme’s active site and all previously identified Ni-binding/catalytically active residues are conserved across all UreCs encoded in our collection [36].

### Sporulation and germination

All clostridial species are able to form metabolically inactive, stress-resistant spores [37]. Sporulation is controlled by the master regulator *spo0A*, a transcriptional regulator which controls expression of sporulation genes [38]. As expected, *spo0A* is present in *C. sordellii* ATCC9714 (ATCC9714_26501). In *Bacillus subtilis* the proteins Spo0F and Spo0B form a phosphorelay from the kinase KinA, resulting in downstream Spo0A phosphorylation and activation [38]. *C. difficile* lacks the *spo0F* and *spo0B* genes, and Spo0A is instead directly phosphorylated by two histidine kinases: CD630_1579 and CD630_2492 [39]. Two homologues of CD630_1579 have been identified in ATCC9714: ATCC9714_07111 and ATCC9714_16961, suggesting a similar mode of regulation and activation of Spo0A in *C. sordellii* as in *C. difficile*.

The majority of the sporulation machinery appears to be conserved between *C. difficile* and *C. sordellii*, and it is likely that the sporulation process in *C. sordellii* is similar to that in *C. difficile*. Also highly similar to *C. difficile* are the spore-coat proteins found in *C. sordellii*. 8 proteins have been confirmed as constituents of the spore-coat in *C. difficile* strain 630: CotABDEFG, CotCB and SodA [40]. Likely homologues have been identified in *C. sordellii* ATCC9714 of CotA (ATCC9714_1722), CotB (ATCC9714_05091), CotD (ATCC9714_05851), CotE (ATCC9714_12131), and SodA (ATCC9714_20791). No specific homologues have been identified for CotCB, CotF or CotG; however, CotCB and CotG are highly similar to CotD and are both also closely homologous to ATCC9714_05851. CotF is homologous to ATCC9714_05861, though this gene appears more closely related to the *C. difficile* gene CD630_2400, currently annotated as the putative spore-coat gene, *cotJB2*, which is very similar to *cotF* but is not yet uncharacterised. It thus seems likely that the *C. sordellii* spore-coat is similar to that of *C. difficile*, but potentially simpler; where *C. difficile* possesses 2 or 3 paralogues of certain genes (*cotCB, cotD* and *cotG*, or *cotF* and *cotJB2*), *C. sordellii* possesses only one equivalent gene. One potentially significant difference between *C. difficile* and *C. sordellii* spores has been identified. *C. difficile* 630 has three *bclA* genes, homologues of which in *B. anthracis* encode glycoproteins forming filaments on the surface of the spore [41]. No such homologues were identified in *C. sordellii* ATCC9714.

Spores are able to germinate and produce vegetative cells. Germination of both *C. difficile* and *C. sordellii* spores is known to be initiated by spore surface receptors that recognise and bind mammalian bile salts [42]. The only known bile salt receptor in *C. difficile* is the serine protease CspC [43], a homologue of which is also found in *C. sordellii* ATCC9714 (ATCC9714_11661).

## Discussion

Our genomic analysis of a collection of *C. sordellii* strains reveals several important findings related to the phylogeny of the species and to the pathogenesis of disease. Phylogenetic analysis reveals the presence of four clades within our strain collection, which includes strains from diverse geographical locations and from infections of both veterinary and medical origin. None of the four assigned clades is specifically associated with either clinical or veterinary disease; rather, both clinical and veterinary isolates are found in each, suggesting that no group of strains has adapted to any specific host. Also, no clade is specifically associated with any country of origin. Given the relative geographical isolation of the USA, UK and Australia, one might have expected strains located in each nation to have diverged. The fact that strains sourced from each country are closely related to strains sourced from the others may suggest recent transfer of strains between countries. How this has occurred remains unclear, though one likely possibility is through human activity such as travel or export of livestock.

We found that only a small minority of *C. sordellii* strains contain the LCC genes *tcsL* and *tcsH*. A similar but smaller study in 2006 resulted in similar findings, with only 1 of 14 isolates analysed containing *tcsL* and none containing *tcsH* [16]. In our study, 5 out of 44 strains encode *tcsL*, though two of those (JGS444 & JGS445) are highly related, suggesting they may in fact be the same strain collected from different sources, possibly during a minor outbreak, and only one carries *tcsH*. The 44 strains sequenced here provide a likely representation of the species as a whole, suggesting that only a minority of strains isolated carry *tcsL* and that *tcsL*^+^/*tcsH*^+^ strains are rare. In support of this, a recent study of 52 isolates from a single US strain collection found only 3 to contain *tcsH* (by PCR), although the number containing *tcsL* was not reported [44].

It is highly relevant that the LCC genes are plasmid encoded. Strain UMC164 was previously shown to carry *tcsL*, and indeed to produce the toxin [16], but the isolate we analysed lacks *tcsL* and the other PaLoc genes. This suggests that the original isolate of UMC164 carried a pCS1-type plasmid but this was lost during sub-culturing. Indeed, in a recent paper describing clinical cases of *C. sordellii* infection, Bouvet *et al.* [45] speculated that the LCC genes are encoded on a mobile genetic element which is lost upon sub-culture. They report isolating strains of *C. sordellii* lacking the *tcsL* gene from clinical cases where toxin activity had been demonstrated in intestinal contents. It is therefore likely that the majority of *C. sordellii* infections are due to strains containing a PaLoc on pCS1-type plasmids, but that these genetic elements are often lost from strains during subculture. Thus the PaLoc and its associated plasmid may be present in a considerably larger proportion of naturally occurring *C. sordellii* strains than indicated by previous studies and indeed our own genome survey. The availability of the DNA sequences for the plasmid and toxin genes will allow early analysis of clinical samples by PCR which could identify toxin genes before they are lost upon subculture.

A previous study employed genetic inactivation of *tcsL* in ATCC9714 to probe the requirement of TcsL to establish disease in two mouse models of infection [13]. In both models the wild type strain caused severe disease while the *tcsL* mutant was avirulent, demonstrating that TcsL is an essential virulence factor in these models, and other studies have also indicated that TcsL is required for the establishment of toxic shock associated with severe *C. sordellii* infections, though non-toxigenic strains are capable of causing less severe infections [14, 15]. Given that the majority of strains in our collection are from clinical/veterinary cases of disease, it seems likely that a significant number of them might have originally harboured a pCS1-type plasmid, which has been lost upon laboratory subculture. This would include UMC164 (as stated above) and any which were associated with severe infections/toxic shock syndrome. From our phylogenetic tree, strain R15892 has probably lost a pCS1-type plasmid, being closely related to the *tcsL*^*+*^ strains ATCC9714 and JGS444/445. It is a matter of speculation why some strains lose their toxin-encoding plasmid while others don’t. Possibilities include the pCS-1 plasmids being stable variants of the highly unstable plasmids or the unstable plasmids having a distinct replication or partition apparatus conferring instability under laboratory conditions.

We should not assume that all strains lacking the PaLoc were once toxigenic and have since lost their pCS1-type plasmids. The ‘UMC’ strains were obtained from cadavers and had not caused infection in their host humans [16], meaning some of them at least might be avirulent. It is also possible that some of the strains described here may have caused infections despite lacking *tcsL*. It has been estimated that only two-thirds of reported cases of *C. sordellii* infection are associated with toxic shock syndrome, and this could even be an overestimate with less dramatic cases being overlooked [14]. At least one of the non-toxigenic strains, DA-108, was derived from a clinical infection not associated with toxic shock syndrome [15], while strain W10 was isolated from a veterinary infection but appears to carry a pCS1-4-like plasmid; i.e. it still carries a pCS1-type plasmid but lacks *tcsL*. All strains in our study contain other putative virulence factors including the cholesterol-dependent cytolysin sordellilysin (*sdl*), neuraminidase (*nanS*), phospholipase C (*csp*) and collagenase (*colA*). Cases of *C. sordellii* invasive disease not linked to toxic shock syndrome and caused by apparently LCC-negative strains have been described [14, 15, 46, 47]. It is therefore possible that a number of these strains were non-toxigenic at the time of infection and were associated with less severe disease (e.g. R30684 was isolated from an abscess).

In *C. difficile*, the toxin genes *tcdA* and *tcdB* are chromosomally localised and strains carrying both toxin genes *tcdA* and *tcdB* constitute the majority of strains isolated from *C. difficile* infections, with those carrying only *tcdB* comprising around 5-10 % of clinical isolates [48, 49]. Non-toxic *C. difficile* strains are routinely isolated from patients [49], but it remains to be demonstrated that such *tcdA*^*−*^/*tcdB*^*−*^ strains are pathogenic. Interestingly, to date no naturally occurring *C. difficile tcdA*^+^/*tcdB*^−^ strains or *C. sordellii tcsH*^+^/*tcsL*^*−*^ strains have been found. The relative orientations of the *C. sordellii* toxin genes and their regulatory genes *tcsE* and *tcsR* are distinct to those found in *C. difficile*, while no homologue of *tcdC,* which negatively regulates toxin production in *C. difficile* [18], is present.

Our findings raise the question of the evolution of the LCC genes. Our phylogenetic analysis suggests that a pCS1-like plasmid has entered strains of *C. sordellii* on at least 3 occasions (see Fig. 1). One possible chronology is as follows: pCS1-3, bearing both *tcsL* and *tcsH*, first entered *C. sordellii* (indicated by “1” in Fig. 1). On a second occasion, “2”, pCS1-3 entered into the ancestor strain of clade 1, which includes ATCC9714. Herein a rearrangement occurred resulting in the loss of the vast majority of *tcsH* and the eventual formation of pCS1-1 and pCS1-2. On a third occasion, “3”, pCS1-3 could have entered an ancestor of strains UMC2 and W10 and undergone a rearrangement resulting in the loss of the entire PaLoc, forming pCS1-4. Of course, several other potential chronologies exist, and given the possibility that multiple strains may have once carried such a plasmid and since lost it this analysis may be lacking. Whole genome sequencing of other known *tcsL*^*+*^ strains would allow improvement of this tree and greater understanding of the distribution of these plasmids throughout the species.

Another question is the origin of the LCC genes in *C. sordellii*, and whether the plasmid bearing them is passed directly between strains of *C. sordellii* or is transferred via an external intermediate. It has recently been shown that *C. difficile* LCC genes can undergo horizontal transfer between strains, a process likely mediated via conjugative transposons [50], but it is not yet known whether the LCC genes of *C. sordellii* can be transferred between strains via plasmid transfer.

## Conclusion

Our sequencing of *C. sordellii* genomes reveals a surprising deficit of LCC genes, that where present they are plasmid-localised, and evidence of plasmid loss from certain strains. This suggests that the LCC genes may be more widespread within the species than the screening of isolates initially indicates. Less severe infections may be caused by LCC-negative strains where virulence is facilitated by other virulence factors, some of which we identified in strain ATCC9714. Although a clade structure was identifiable in our collection of strains, these did not divide by geographical region or by infected host species. The high-quality reference genome sequence of *C. sordelllii* ATCC9714 will enable further research on this important pathogen. Finally, comparison of *C. sordelllii* sequences with those of other pathogenic clostridia will help to characterise the wide range of strategies that the clostridia use to survive and proliferate in man and animals.

## Methods

### Bacterial strains and growth conditions

*C. sordellii* strains were grown in BHIS broth (37 g/L Brain-Heart Infusion, 5 g/L yeast extract, 1 g/L L-Cysteine) or on BHIS agar (as above, with 15 g/L agar) in an anaerobic cabinet (Don Whitley Scientific) in an atmosphere of 80 % N_2_, 10 % CO_2_ and 10 % H. A full list of strains used in this study can be found in Table 1.

### DNA extraction and sequencing

Genomic DNA was prepared by growth of 25 ml overnight cultures of *C. sordellii* in BHIS broth. Cells were harvested by centrifugation (4000 x g, 10 min). Cells were frozen at -80 °C for 1 h then re-suspended in 400 μl lysis buffer (200 mM NaCl, 50 mM EDTA, 20 mM Tris–HCl, pH 8). Lysozyme (2 mg/ml) and RNase A (0.1 mg/ml) were added and the suspension incubated at 37 °C for 2 h. Proteinase K (0.5 mg/ml) and SDS (1 %) were added and the suspension incubated at 50 °C for 1 h. DNA was then extracted using phenol/chloroform, and precipitated by adding 2.5 volumes of ice-cold ethanol followed by overnight incubation at -20 °C. DNA was harvested by centrifugation at 17 900 x g for 5 min at 4 °C, washed with 1 ml 70 % ethanol, centrifuged at 17 900 x g for 5 min at 4 °C again and the supernatant removed. The pellet was air-dried at room temperature then resuspended in H_2_O. Genomic DNA was sequenced on an Illumina Hi-Seq in a multiplex run of 48 samples.

### Assembly of genomes and annotation

*De novo* assembly of the Illumina sequence data into contigs was carried out using VelvetOptimiser (Victorian Bioinformatics Consortium). The genome of ATCC9714 was then optically mapped [51] (see below), establishing the order and relative orientations of all contigs >50 kb. Gapfiller [52] (BaseClear) was then used to attempt to computationally close sequence gaps and iCORN (http://icorn.sourceforge.net) to correct for any sequence errors. CDSs (Coding DNA Sequences) were identified using Prodigal [53] and functional annotation was transferred using four other clostridial genomes as references in an iterative process: *C. difficile* 630 (accession number AM180355); *Clostridium botulinum* Hall A (Proteolytic) (accession number AM412317); *C. botulinum* E3 Strain Alaska E43 (non-proteolytic) (accession number CP001078); *Clostridium novyi* NT (accession number CP000382) (in that order). The genome sequence was further improved using a combination of manual inspection of sequence data, PCR and comparison to the other *C. sordellii* strains and other clostridia. (For primer sequences, see Additional file 2). Subsequent mapping of the sequence data to the optical map allowed for validation of the genome assembly. Annotation to identify coding sequences, assign predicted functions and to predict RNA structures of the other *C. sordellii* strains was generated using Prokka [54].

### Optical mapping

BglII was identified by OpGen enzyme selection software as an appropriate restriction enzyme with which to digest *C. sordellii* gDNA. 2 μl gDNA was applied to an OpGen MapCard and run on an Argus® system following manufacturer’s protocols. The MapCard chambers were loaded with JOJO™ stain and OpGen enzyme, buffer and antifade, and the card cycled on the Argus® MCP (MapCard Processing Unit) for approximately 25 min at 37 °C. Following data collection, contig assembly was performed using Argus® MapManager™; prior to assembly the mapset was filtered for minimum molecule size >250 kb, minimum fragments per molecule >12 and minimum molecule quality >0.4. The genome was initially assembled into a contiguous, circularized chromosome. Any regions of low coverage were improved using the "Find Hits" feature until depth of coverage reached 30x. The QC module was then used to perform a QC review to ensure all regions of map assembly were robust. The *de novo* sequence assembly was then aligned to the Optical Map using Argus Mapsolver. 89.8 % of the *de novo* sequence assembly was aligned to the Optical Map with the remainder either contigs smaller than 50 kb which were too small to accurately align or plasmid sequence which was not incorporated into the Optical Map assembly.

### Generation of phylogenetic tree

A core genome of *C. sordellii* was created using CD-HIT [55] to perform an initial clustering of the common genes shared between all 44 strains based on a 90 % identity threshold followed by an all-against-all blast, the results of which were then input to a Markov Cluster Algorithm to perform the final clustering. In total 2712 genes were clustered to create the core *C. sordellii* genome. The sequences were concatenated and a nucleotide alignment was created using Muscle [56]. A maximum likelihood phylogeny was generated from the aligned sequence using FastTree version 2.1.3 [57] with the following settings: a Generalised Time-Reversible (GTR) model of nucleotide substitution and CAT approximation of the variation in rates across sites with 20 rate categories. BLASTN queries of the assembled *C. sordellii* genomes along with reciprocal annotation using different members of the order Clostridiales against the *C. sordellii* ATCC9714 genome revealed *C. difficile* to be the closest relative (86 % BLASTN identity). *C. difficile* proved to be too diverse as an out-group to determine a meaningful phylogeny with *C. sordellii* using the methods described above. The root of the tree was therefore established by aligning the 7 MLST genes [58] of the *C. difficile* strain R20291 (Ribotype 027, Accession no. FN545816) with their corresponding *C. sordellii* orthologues (all present in every strain at 90 % identity) and generating a phylogeny from the alignment using FastTree as described above. R20291 was chosen as it was the closest match to *C. sordellii* with a high-quality sequence.

### PCR

PCR reactions were performed with KOD Hot Start DNA polymerase (Merck Millipore). Reactions were set up according to the manufacturer’s instructions. 200 ng of gDNA was used in each reaction which entailed an initial denaturation step (94 °C, 2 mins) followed by 30 cycles of: 94 °C, 15 s; primer Tm minus 5 °C, 30 s; 68 °C, 1 min/kb predicted product size. The primer Tms were calculated using https://ecom.mwgdna.com/services/webgist/mops.tcl. PCR products were purified using a Qiagen QIAquick® PCR Purification Kit. Screening for *tcsL* was performed using primers NF2362 and NF2363 and for *tcsH* using NF2351 and NF2352.

### Pulsed-field gel electrophoresis (PFGE)

Growth of *C. sordellii* for pulsed-field electrophoresis analysis was conducted at 37 °C in an atmosphere of 10 % H_2_, 10 % CO_2_ and 80 % N_2_ in an anaerobic chamber (Coy Laboratory Products, Inc). *C. sordellii* was grown on Nutrient Agar (25 g/L Nutrient Broth No. 2 (Oxoid), 3 g/L yeast extract, 1 g/L sodium thioglycolate, 15 g/L agar, 0.375 % glucose) to obtain single colonies prior to growth overnight in 20 mL BHI broth (35 g/L Bacto Brain Heart Infusion (BD), 1 g/L sodium thioglycollate, 0.375 % glucose). Purified *C. sordellii* genomic DNA was extracted from 5 mL of overnight BHI culture as previously described [59] with the omission of RNAse upon resuspension of DNA. To produce agarose plugs containing *C. sordellii* genomic DNA, overnight BHI cultures were used to inoculate fresh 20 ml BHI broths to an OD_600_ of 0.1. These broth cultures were then grown to an OD_600_ of ~1.0. The bacterial cells were pelleted from 10 ml of culture at 2466 x g for 8 min. Bacterial cells were washed in 10 ml PIV buffer (10 mM Tris–HCl, 1 M NaCl, pH 7.6) and pelleted at 2466 x g for 8 min. Cells were resuspended in 1 ml PIV buffer and mixed with an equal volume of 1.5 % SeaPlaque® Agarose (Lonza) in 0.5 x TBE buffer (the agarose having been equilibrated to 50 °C). The cell-containing agarose plugs were cast in 1.5 mm-thick moulds (Bio-Rad) at room temperature (RT). The plugs were removed from the moulds and incubated at 37 °C for 2 h with gentle shaking in lysis buffer (0.5 M EDTA [pH 8], 0.5 % Sarkosyl, 2.5 mg/ml lysozyme, 2 mg/ml deoxycholic acid). The plugs were then incubated in proteinase K buffer (0.5 M EDTA [pH 8], 0.5 % Sarkosyl, 1 mg/ml proteinase K) at 50 °C overnight. Plugs were then washed 4 x 15 min in 0.1 x TE, followed by washing in 1 x TE, all washing occurring at RT with gentle shaking. Plugs were stored in fresh 1 x TE at 4 °C. To linearise pCS1-type plasmids so as to ensure more accurate size comparisons, agarose plugs used in this experiment were incubated with or without restriction enzyme *Blp*I (New England Biolabs) in 450 μl NEBuffer 4 (New England Biolabs). PFGE of both extracted genomic DNA and agarose gel plugs containing genomic DNA were performed using a 1 % Pulsed Field Certified Agarose (Bio-Rad) gel in 0.5 x TBE buffer using the CHEF-DR III System (Bio-Rad) at 15 °C. A Mid-Range I PFG Marker (New England Biolabs) was used as a molecular size standard. Electrophoresis parameters were as follows: 6 V/cm, ramping pulse from 1 to 25 s for 25 h. Gels were stained in GelRed (Biotium) and photographed under UV light.

### Southern Hybridisation

DNA from pulsed-field gels was transferred to a nylon membrane (Roche) as previously described [60] with the following modifications: 2 × 20 min incubations in denaturation solution, 2 × 20 min incubations in neutralisation solution and a 48 h transfer. The blots were hybridised to a *tcsL*-specific PCR product amplified using the primers DLP236 and DLP237, a pCS1 *parB*-specific PCR product amplified using the primers DLP377 and DLP378 or a ATCC9714PCS2_00141-specific PCR product amplified using the primers DLP362 and DLP363. All probes were amplified from *C. sordellii* ATCC9714 genomic DNA. Probes were digoxigenin-labelled using random PCR labelling according to the manufacturer’s instructions (Roche). Hybridisation was detected using the CDP-Star (Roche) chemiluminescence detection system according to the manufacturer’s instructions.

### Availability of supporting data

The sequences described here have been deposited in the NCBI/EBI databases. Accession numbers for the genome of each strain are given in Table 1. These are accessible through the WTSI website (http://www.sanger.ac.uk/resources/downloads/bacteria).

## Additional files

Additional file 1:
**Putative Secreted Proteins of** ***C. sordellii*** **strain ATCC9714.** Herein is contained a list of all putative secreted proteins encoded by the genome of *C. sordellii* ATCC9714, as identified by SignalP and Phobius, identifying them by gene number and indicating if they are proposed to be lipoproteins, if they are annotated with a specific gene product and giving a putative function based on BLAST analysis. Additional file 2:
**Primers Used in This Study.** Herein is contained a list of all primers used in this study, giving their name/number, sequence and what they were used for.

## Abbreviations

LCC: Large clostridial cytotoxin
PFGE: Pulse field gel electrophoresis
