# Supplementary material for: Clostridium sordellii genome analysis reveals plasmid localized toxin genes encoded within pathogenicity loci
Source: BMC Genomics. 2015 May 16;16(1):392. doi: 10.1186/s12864-015-1613-2 (PMC4434542; doi:10.1186/s12864-015-1613-2)
Supplement: Additional file 1: — Putative Secreted Proteins of C. sordellii strain ATCC9714. Herein is contained a list of all putative secreted proteins encoded by the genome of C. sordellii ATCC9714, as identified by SignalP and Phobius, identifying them by gene number and indicating if they are proposed to be lipoproteins, if they are annotated with a specific gene product and giving a putative function based on BLAST analysis. [file 12864_2015_1613_MOESM1_ESM.pdf]

C. sordellii ATCC 9714 Secreted Proteins

Putative Secreted Proteins (SignalP/Phobius)

Gene Number	Putative Lipoprotein?	Gene Product (If Annotated)	Putative Function
9714_00931	Y		ABC transporter amino acid-binding protein.
9714_01031	N		Unknown.
9714_01151	N	PilA1C	Type IV pilin.
9714_01231	Y	PrsA	Catalyses post-translocational folding of exported proteins.
9714_01791	Y		OppA - Oligopeptide-binding component of ABC Transporter.
9714_01801	Y		OppA - Oligopeptide-binding component of ABC Transporter.
9714_02601	N		Unknown.
9714_02791	N	SecG	Sec translocase component.
9714_03151	N		Unknown - Cell Wall-Binding (3 x SLH domains)
9714_03161	N		Unknown.
9714_03211	Y		Metal-binding component of an ABC transporter.
9714_03241	Y		Unknown.
9714_03251	Y		Sulphur Transferase.
9714_04221	Y		Unknown.
9714_04271	Y		ABC transporter solute-binding component.
9714_04351	N	RnfG	Electron transport chain component.
9714_04721	N	DacF1	D-alanyl-D-alanine carboxypeptidase. Penicillin-binding protein.
9714_04851	Y		ABC transporter component.
9714_04901	N	SpoIIP	Stage 2 sporulation protein P. Autolysin with peptidoglycan hydrolase activity.
9714_05111	N		Unknown.
9714_05181	Y	PotD1	Spermidine/putrescine-binding component of ABC transporter.
9714_05681	Y	PstS	Phosphate-binding component of phosphate-specific transport system.
9714_05831	N		Cell wall hydrolase.
9714_05971	N		M23 Peptidase.
9714_06101	N		Unknown.
9714_06251	N		Unknown.

9714_06291	N		Penicillin-binding transcriptional regulator.
9714_06351	N	DacF2	D-alanyl-D-alanine carboxypeptidase. Penicillin-binding protein.
9714_06571	N		Unknown.
9714_06661	N		Iron-binding component of ABC transporter .
9714_06711	N	EntD	Mannosyl-glycoendo-beta-N- acetylglucosamidasedomain protein.
9714_07231	Y		Iron-binding component of ABC transporter.
9714_07381	Y		Membrane-associated nucleotidase.
9714_07391	Y		Membrane-associated nucleotidase.
9714_07441	Y		Ferrichrome binding component of ferrichrome transporter.
9714_07511	N		Unknown.
9714_07731	N		Unknown.
9714_07751	N		Hydrolase.
9714_07781	Y		Glycoside hydrolase.
9714_08171	N		Unknown.
9714_08211	N		OppA - Oligopeptide-binding component of ABC Transporter.
9714_08261	Y		OppA - Oligopeptide-binding component of ABC Transporter.
9714_08311	N		Transglycosylase.
9714_08671	N		Amidase.
9714_08711	N	KdpC	Potassium-transporting ATPase C chain.
9714_08911	N		Transglycosylase.
9714_08921	N		Transglycosylase.
9714_09041	N		Unknown.
9714_09321	Y	MtnN2	5'-methylthioadenosine/S-adenosylhomocysteine nucleosidase.
9714_09441	Y		Unknown.
9714_09531	N		Unknown.
9714_09551	N		Unknown.
9714_09631	N		Unknown
9714_09671	N		Unknown.
9714_09771	N		Unknown - Cell Wall-Binding (3 x SLH domains)
9714_09781	N		Unknown - Cell Wall-Binding (3 x SLH domains)
9714_09801*	N		IgG Specific Protease - Cell Wall-Binding (3 x SLH domains)
9714_09831	Y		Unknown.
9714_10021	Y		Unknown.

9714_10061*	N	ColA	Collagenase.
9714_10411	N		Unknown.
9714_10741	N		Unknown.
9714_10771	Y		Unknown.
9714_10821	N		Unknown.
9714_10871	Y		Unknown.
9714_10931	N		Methyl-accepting chemotaxis sensory transducer.
9714_10941	N		Unknown.
9714_11121	N		Unknown.
9714_11511	N		Cell wall hydrolase.
9714_11521	N		Unknown.
9714_11571	Y		Penicillin-binding protein.
9714_11631	N		Unknown.
9714_11681	N		Unknown.
9714_12241	N		Polysaccharide deacetylase.
9714_12921	N		Glycoside hydrolase.
9714_13041	Y		Rhodanese.
9714_13051	Y		ABC transporter substrate-binding component.
9714_13361	N		S41 Peptidase.
9714_13371	N		Unknown.
9714_13591	Y		Unknown.
9714_13631	Y		Unknown.
9714_13801*	N	Aureolysin	Metalloprotease.
9714_13981	Y		ABC transporter substrate-binding component.
9714_14151	Y		Unknown.
9714_14191	N		Unknown.
9714_14211	Y		Unknown.
9714_14311	N		Unknown.
9714_14451	Y		Unknown.
9714_14471	N		Unknown.
9714_14621	Y		S41 Peptidase.
9714_15101	Y		ABC transporter substrate-binding component.
9714_15361	Y		Ribose-binding component of an ABC transporter.

9714_15681	Y		Ferrichrome/iron binding component of an ABC transporter.
9714_15841	Y		C4-dicarboxylate transporter.
9714_15951	N		Unknown.
9714_16161*	Y	NanS	Neuraminidase.
9714_16431	N		Unknown.
9714_17781	Y	RbsB	Ribose-binding component of an ABC-transporter.
9714_17991	N		Unknown.
9714_18161	Y		Unknown.
9714_18311*	N	SdlO	Cholesterol-dependent cytolysin.
9714_19591	Y		Unknown.
9714_19641	Y		Unknown.
9714_19651	Y		Unknown.
9714_19671	N		Unknown - Cell Wall-Binding (3 x SLH domains)
9714_20171	Y		Unknown.
9714_20421	N		Metal protease.
9714_20601	N		Unknown.
9714_20661	Y	FhuD	Ferrichrome-binding component of ABC transporter.
9714_20871	Y		β-Lactamase
9714_21301	N		Unknown.
9714_21601	Y		ABC transporter substrate-binding component.
714_21751/2176	N		Amidase.
9714_21821	N		Phosphoenolpyruvate:sugar phosphotransferase system component.
9714_21921	N		Unknown.
9714_22051	Y		Unknown.
9714_22371	Y		Iron-binding component of an ABC transporter.
9714_22391	N		Unknown.
9714_22621	N	CbiM	Cobalamin synthesis protein.
9714_22651	Y		Iron-binding component of an ABC transporter.
714_22821/2283	N		Collagen Binding Protein.
9714_23021	N		Unknown.
9714_23421	Y		Sporulation protein.
9714_23451	N		Unknown.
9714_23561	Y		Unknown.

9714_23681	N		D-alanyl-D-alanine carboxypeptidase.
9714_23951	Y		Unknown.
9714_23971	Y		Unknown.
9714_24071	Y		Carboxypeptidase
9714_24101	N		Unknown.
9714_24181	N		M23 Peptidase.
9714_24341	Y		Polysaccharide deacetylase.
9714_24381	N		Unknown.
9714_24461	Y		Unknown.
9714_24811	N	FlgK	Flagellar Hook-Associated Protein.
9714_24971	Y		Unknown.
9714_25311	N		Unknown.
9714_25621	N		Unknown.
9714_25631	N		Unknown.
9714_25761	N		Unknown - Cell Wall-Binding (3 x SLH domains)
9714_25881	Y		Nitrate/sulphonate/taurine binding component of an ABC transporter.
9714_25891	N		Unknown.
9714_26621	N	SpoIIIAH	Stage III sporulation protein AH.
9714_26651	N	SpoIIIAE	Stage III sporulation protein AE.
9714_27551	N	SpoVD	Stage V sporulation protein D (Sporulation-specific penicillin-binding protein).
9714_27871	N		Unknown - Cell Wall-Binding (3 x SLH domains)
9714_28071	N		Unknown - Cell Wall-Binding (3 x SLH domains)
9714_28081	N		Amidase - Cell Wall-Binding (3 x SLH domains)
9714_28661	N		Transglycosylase.
9714_29321	Y		Unknown.
9714_30341	Y		Unknown.
9714_30571	N		Cell wall hydrolase.
9714_30891	N	SpoIIC	Stage II sporulation protein D (or C!?!).
9714_30911	N		Unknown.
9714_31131	N		Polysaccharide deacetylase.
9714_31171	N		Spore cortex lytic enzyme.
9714_31321*	N	PlC	Phospholipase C
9714_31871	N		Lipoate-protein ligase.

9714_32021	Y	PotD2	Spermidine/putrescine-binding component of ABC transporter.
9714_32111	Y		Unknown.
9714_32231	N		ABC transporter substrate-binding component.
pCS1_00191	Y		Sporulation protein.
pCS1_00241*	N		Collagen adhesion protein.
pCS1_00531	N		Unknown.
pCS1_00791	N		Unknown.
pCS1_00831	N		Unknown.
pCS1_00871	N		Unknown.
pCS2_00021	Y		Unknown.

* indicates gene product is putative virulence factor
