# Supplementary material for: Clostridium sordellii genome analysis reveals plasmid localized toxin genes encoded within pathogenicity loci
Source: BMC Genomics. 2015 May 16;16(1):392. doi: 10.1186/s12864-015-1613-2 (PMC4434542; doi:10.1186/s12864-015-1613-2)
Supplement: Additional file 2: — Primers Used in This Study. Herein is contained a list of all primers used in this study, giving their name/number, sequence and what they were used for. [file 12864_2015_1613_MOESM2_ESM.pdf]

Primers Used In Improving *C. sordellii* Genomes (5'-3')

Pair	Primer Pairs Used in Improving ATCC9714 Chromosome Assembly		Tm (^oC)	Genome Binding Location (Nucleotide No.s, 5' - 3')¶	DNA Strand Bound
A	NF2419	ATAAGTTTTAGAATAGATTAAACAATAATAAGGAG	58.9	372640 - 372674	Forward
	NF2420	TTCTAGGCCTATAATAATCAATTGTCG	58.9	377087 - 377061	Reverse
B	NF2425	TTCTACACCTAATGTTACTTGGTTTATAG	59.6	2379678 - 2379706	Forward
	NF2426	TCAATAGAAGAATTATCTGCAACTATAATGG	60.2	2380211 - 2380181	Reverse
C	NF2467	TTCATAGTTTAAATTAAATTCAACTTGATCTC	58	2359824 - 2359855	Forward
	NF2468	TTAGATAAGGTGGTAATAATGAAAACG	57.6	2361923 - 2361897	Reverse
D	NF2533	AAGCAACTGGTACAAATACTATAGC	58.1	374143 - 374119	Reverse
	NF2534	AGTTTCAATACTAGAAAGTGGAACATC	58.9	375225 - 375251	Forward
E	NF2695	TAGCTACAAGTATAATAACTGATCC	56.4	29025 - 29049	Forward
	NF2696	TACACCTCCAACTCGTAAATAC	56.5	30741 - 30720	Reverse
F	NF2697	CTTCAGTTGCTAAGAAAATAGCTG	57.6	44993 - 45016	Forward
	NF2698	TCTTCTTTTAAGCCTCTTGTCAAC	57.6	46454 - 46431	Reverse
	**Primers Used to Assemble ATCC9714 Plasmid pCS1-1**				
G	NF2409	ACATATATTGAAATGTTTTAATTTATATATTTTGTG	56.9	46021 - 45986	Reverse
	NF2410	TATTAAAAGAAGTATTTGAAGGTCAAATATC	57.6	45473 - 45503	Forward
H	NF2411	TACAAAGGAACTGAAAATCTATTTCG	56.9	43582 - 43557	Reverse
	NF2412	ATATATTCAAAGGAGAGCAAATAGAG	56.9	43153 - 43178	Forward
I	NF2413	ACAGTAGATGATAGTAAAGTAGATTGG	58.9	100766 - 100740	Reverse
	NF2414	GATAGTATAAAGTTTAGTACTTCTAAATCG	58.6	98918 - 98947	Forward
J	NF2415	ATGGAATAACATAACGAAAGATATTAATG	56.8	92808 - 92780	Reverse
	NF2416	ATTTATTACAGGCATACTATTTATTACAAC	57.2	91401 - 91430	Forward
K	NF2417	CTAAAGATACTAGTGAAGATATATATGC	57.8	83927 - 83900	Reverse
	NF2418	ACAAAATATAAAGCTATTCCACTATCAC	57.8	83429 - 83456	Forward
L	NF2634	TAGTTTCATCAGATAGATTATTATGTTG	56.3	79941 - 79968	Forward
	NF2635	TAAGTATACCTAATCCTATAATTGATAC	56.3	81692 - 81665	Reverse

Primers Used to Assemble ATCC9714 Plasmid pCS2

M	NF2632	AGTAAGCTTGATAAATTTAGTGAAGG	56.9	22232 - 22207	Reverse
	NF2633	TTGTTCTAAATTAACACCTAAATCTTTAG	56.8	21690 - 21718	Forward

Primers Used to Assemble JGS6364 Plasmid pCS1-2

G	NF2409	ACATATATTGAAATGTTTTAATTTATATATTTTGTG	56.9	45963 - 45928	Reverse
	NF2410	TATTAAAAGAAGTATTTGAAGGTCAAATATC	57.6	45415 - 45445	Forward
H	NF2411	TACAAAGGAACTGAAAATCTATTTCG	56.9	43524 - 43499	Reverse
	NF2412	ATATATTCAAAGGAGAGCAAATAGAG	56.9	43095 - 43120	Forward
I	NF2413	ACAGTAGATGATAGTAAAGTAGATTGG	58.9	114263 - 114237	Reverse
	NF2414	GATAGTATAAAGTTTAGTACTTCTAAATCG	58.6	112415 - 112444	Forward
J	NF2415	ATGGAATAACATAACGAAAGATATTAATG	56.8	106305 - 106277	Reverse
	NF2416	ATTTATTACAGGCATACTATTTATTACAAC	57.2	105153 - 105182	Forward
K	NF2417	CTAAAGATACTAGTGAAGATATATATGC	57.8	97679 - 97652	Reverse
	NF2418	ACAAAATATAAAGCTATTCCACTATCAC	57.8	97262 - 97289	Forward
L	NF2634	TAGTTTCATCAGATAGATTATTATGTTG	56.3	93776 - 93803	Forward
	NF2635	TAAGTATACCTAATCCTATAATTGATAC	56.3	95527 - 95500	Reverse
N*	NF3067	TAAGGATTTTACTTCATCTACTGTCAATC	59.6	56750 - 56778	Forward
	NF3068	CTCCTTGATAAATAGAATCTATATCTTCTTC	60.2	58453 - 58447	Reverse
O	NF3068	CTCCTTGATAAATAGAATCTATATCTTCTTC	60.2	78496 - 78466	Reverse
	NF3073	TTTGGATTATGGCGAGAAATTTTATTTGC	59.6	77808 - 77836	Forward

Primers Used to Assemble JGS6382 Plasmid pCS1-3

P	NF2413	ACAGTAGATGATAGTAAAGTAGATTGG	58.9	101540 - 101514	Reverse
	NF2699	CAAAGTGAACTACTTATAAATCTAATAAATTTC	58.3	99166 - 99198	Forward
Q	NF2700	ATAGAGATGGATCCTCAGTTAATG	57.6	92585 - 92562	Reverse
	NF2701	ATTTGTATTCCCAGGTGGTTTAG	57.1	90628 - 90650	Forward
R	NF2702	TAGAGTTTTCTAGGGTTCCGTAG	58.9	74280 - 74258	Reverse
	NF2703	GAAGTAGTAAAAAAGCCCTTAAAATCC	58.9	72756 - 72782	Forward

	**Primers Used to Assemble UMC2 Plasmid pCS1-4**				
**S**	NF2634	TAGTTTCATCAGATAGATTATTATGTTG	56.3	80698 - 80725	Forward
	NF3057	TATTTCAGGGCTAACAAATTTAGAC	56.4	81700 - 81676	Reverse
**T**	NF3058	CAGTAATTTTAAGGAGATGAACTATAC	57.4	97974 - 98000	Forward
	NF3059	AGTGACAGGTCTTATGGCTAAC	58.4	416 - 395	Reverse
**U**	NF3060	TGTATCTTCTCGGAATAATTTCTCC	58.1	14110 - 14134	Forward
	NF3061	CTTAAGTTTATGTCATATTTTCTTGGTTC	58.2	16536 - 16508	Reverse
	**Primers Used to Produce Probes for Southern Blots**				
tcsL	DLP236	GATGATAACGGAATAAGGCAATTAGG	60.1	pCS1-1: 10763 - 10788	Forward
	DLP237	AAGCCCTGTTCTCATTATACCATTCTC	61.9	pCS1-1: 11185 - 11159	Reverse
**pCS2**	DLP362	ATGACATATAAAGTAGCAAGGAATCAGATG	61.3	pCS2: 10725 - 10754	Forward
	DLP363	GCCCATAGGTTGCTGCTAGTTTC	62.4	pCS2: 11149 - 11127	Reverse
parB	DLP377	AACTTTCAACTCTTGAGGCAAAGG	59.3	pCS1-1: 1595 - 1572	Reverse
	DLP378	CCTACCTGTGTAGGACTCAGTCCAG	66.3	pCS1-1: 1127 - 1151	Forward
	**Primers Used to Screen for *tcsL* and *tcsH***				
tcsH	NF2351	TGCAGCATCTGATTTAGTAAGG	56.5	pCS1-3: 20072 - 20051	Reverse
	NF2352	TCAAATTGGTATTTTGCGCTTGC	57.1	pCS1-3: 19275 - 19297	Forward
tcsL	NF2362	GGTAAATGGATAAATAAAGAAGAAAG	55.3	pCS1-1: 6701 - 6726	Forward
	NF2363	GATATATGAGTAGCATATTCAGAG	55.9	pCS1-1: 7203 - 7180	Reverse

¶ For clarity, all nucleotide no.s given refer to the forward strand. Because the ATCC9714 chromosome and plasmid pCS1-4 from UMC2 are incompletely assembled, if these assemblies are further improved some of these nucletide no.s may alter.

***** Primer pair N was designed to test a hypothetical assembly which turned out to be incorrect. However, NF3068 serendipitously bound to a non-specific binding site with some similarity to the target site (used in Pair O), producing an unexpected product which allowed us to complete the assembly of plasmid pCS1-2.
